# Targeting RTK Signaling Pathways in Cancer

**DOI:** 10.3390/cancers7030860

**Published:** 2015-09-03

**Authors:** Tarik Regad

**Affiliations:** The John van Geest Cancer Research Centre, School of Science and Technology, Nottingham Trent University, Clifton Lane, NG11 8NS Nottingham, UK; E-Mail: tarik.regad@ntu.ac.uk; Tel.: +44-(0)-115-8483501

**Keywords:** RTK, MAP kinase, PI3K, AKT, small molecule inhibitors, cancer

## Abstract

The RAS/MAP kinase and the RAS/PI3K/AKT pathways play a key role in the regulation of proliferation, differentiation and survival. The induction of these pathways depends on Receptor Tyrosine Kinases (RTKs) that are activated upon ligand binding. In cancer, constitutive and aberrant activations of components of those pathways result in increased proliferation, survival and metastasis. For instance, mutations affecting RTKs, Ras, B-Raf, PI3K and AKT are common in perpetuating the malignancy of several types of cancers and from different tissue origins. Therefore, these signaling pathways became prime targets for cancer therapy. This review aims to provide an overview about the most frequently encountered mutations, the pathogenesis that results from such mutations and the known therapeutic strategies developed to counteract their aberrant functions.

## 1. Introduction

**Figure 1:**
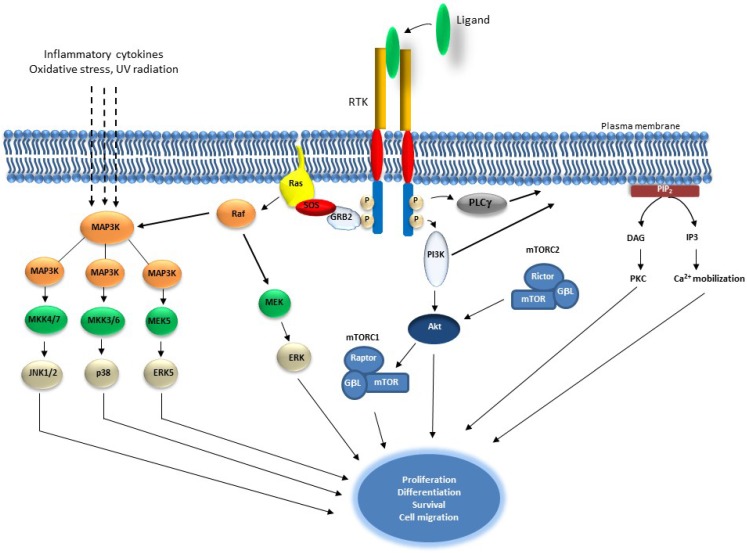
Schematic representation of Receptor Tyrosine Kinase and downstream signaling pathways. Receptor Tyrosine Kinases are auto-phosphorylated upon ligand binding, which results in the activation of Ras and induction of serine/threonine kinase Raf. Raf phosphorylates Mek1/2 which in turn phosphorylate and activate Erk1/2. Raf also activates MAP3 kinases that activate MKK4/7, MKKK3/6 and MEK5, which activates JNK1/2, p38 and ERK5, consecutively. MAP3Ks are also activated by inflammatory cytokines, oxidative stress and UV radiation. PI3K is activated by RTK autophosphorylation and results in the activation of Akt which also induces mTOR within the mTORC1 complex. Akt is also regulated by mTORC2 complex. PLCγ activation leads to Ca^+2^ mobilization and to the activation of PKC. These events play an essential role in proliferation, differentiation, survival and cell migration.

Receptor tyrosine kinases (RTKs) are a family of cell surface receptors, which act as receptors for growth factors, hormones, cytokines, neurotrophic factors and other extracellular signaling molecules. RTKs mediate key signaling pathways that are involved in cell proliferation, differentiation, survival and cell migration [1]. The RTK family comprises several subfamilies which include, among others, epidermal growth factor receptors (EGFRs), fibroblast growth factor receptors (FGFRs), insulin and insulin-like growth factor receptors (IR and IGFR), platelet-derived growth factor receptors (PDGFRs), vascular endothelial growth factor receptors (VEGFRs), hepatocyte growth factor receptors (HGFRs), and proto-oncogene c-KIT [2,3]. RTKs monomers are organized into an extracellular (N-terminal), a transmembrane and a cytoplasmic kinase domain. They are activated via ligand-induced dimerization that results in receptor auto-phosphorylation and tyrosine activation of RTKs’ substrates including phospholipase C-γ, mitogen-activated protein kinases and phosphatidylinositol 3-kinase [4,5,6] (Figure 1).

Mutations that affect RTK signaling often lead to cell transformation, which is observed in a wide variety of malignancies. These mutations affect RTKs or components of downstream pathways such as MAP kinase and the PI3K/AKT. This results in increased cell proliferation, survival, invasion and metastasis. Therefore, targeting RTK signaling pathways remains a challenge for scientists and clinicians working in the cancer field. Several small molecule inhibitors and antibodies are being clinically developed to target RTKs, the MAP kinase and PI3K/AKT pathways. This review attempts to highlight the important role played by RTK signaling in carcinogenesis and the therapeutic strategies available, so far, to target these important cellular pathways.

## 2. Receptor Tyrosine Kinase Signaling and Cancer

### 2.1. Targeting Receptor Tyrosine Kinases (RTKs) in Cancer

**Figure 2:**
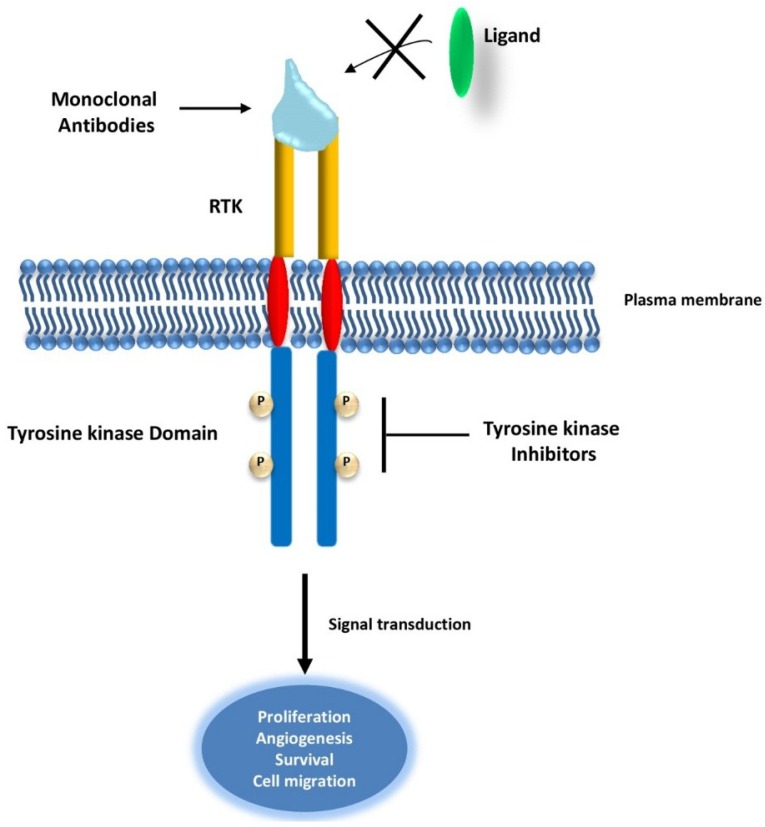
Schematic representation of the mode of action of RTKs inhibitors. In cancer therapy, RTKs are targeted using monoclonal antibodies that prevent ligand binding and therefore the activation of downstream signaling pathways. Tyrosine kinase inhibitors act on the tyrosine kinase domain of RTK, preventing receptors’ auto-phosphorylation and inhibiting signal transduction.

Most RTKs are found mutated in a variety of cancers and from different tissue origins. This chapter discusses the role of RTKs in cancer and the therapeutic strategies developed to target them (Figure 2 and Table 1).

**Table 1:** Examples of RTK targeted molecular cancer therapies being used clinically or subjected to clinical trials.

Target	Compound	Cancer	References
EGFR family			
HER2	Trastuzumab (Herceptin)	HER2-positive breast cancer	[7,8,9]
EGFR	Cetuximab (Erbitux)	Metastatic colorectal cancer (RAS wild type) Metastatic non-small-cell lung cancer	[10]
	Panitumumab (Vectibix)		[10,11,12]
	Gefitinib (Iressa)		[13,14,15]
	Erlotinib (Tarceva)		[13,16]
EGFR and HER2	Lapatinib (Tykerb	HER2-positive breast cancer (Trastuzumab-resistant)	[14,17]
		
	Afatinib	NSCLC HER2-positive breast cancer	[18,19,20,21]
		
VEGFR	Sorafenib (Nexavar)	Renal, liver and thyroid cancer	[22,23,24]
	Sunitinib (Sutent)	Renal cell cancer Gastrointestinal stromal tumor (GIST)	[25,26]
		
	Bevacizumab (Avastin)	Metastatic colorectal carcinoma	[27]
PDGFR	Imatinib (Gleevec)	GIST (KIT+)	[28]
PDGFR and VEGFR	Sunitinib	Angiogenesis	[29,30,31,32]
	Soratinib		
	Pazopanib		
	Nilotinib		
FGFR and VEGFR	Brivanib (BMS-540215)	Human hepatocellular carcinoma model	[33]
VEGFR, PDGFR, FLT-3, c-KIT and FGFR	CHIR-258 (TKI-258)	Multiple myelomas	[34,35]
MET	SGX523	MDCK and A549 cells and GTL16 xenograft models	[36]
C-KIT	Imatinib (Gleevec)	GIST	[37,38,39]

#### 2.1.1. EGFR-Targeted Therapy

Gene mutations affecting EGFR members have been associated with several cancers [40]. In breast cancer, overexpression of HER2 (ERBB2) is found in approximately 10%–30% of patients and is associated with reduced survival [41]. Mutations affecting EGFR gene result in its overexpression in 30%–50% of glioblastomas [42,43], 25%–82% in colorectal cancer [44,45,46,47] and 5%–20% in non-small-cell lung cancer [13,48]. Therefore, molecular targeted therapeutics were developed against those receptors. Trastuzumab (Herceptin), a monoclonal antibody, is used to target the extracellular domain of the HER2 protein in HER2-positive breast cancer patients and has been shown to increase survival at early and late stages of breast cancer [7]. Cetuximab (Erbitux) and Panitumumab (Vectibix) are two other examples of monoclonal antibodies that are used to target the EGFR-ligand binding in the treatment of patients with metastatic colorectal cancer [11,12]. The benefit of cetuximab and Panitumumab was limited to patients with *RAS* wild-type tumors [10].

Lapatinib (Tykerb), a tyrosine kinase inhibitor, targets the ATP binding pocket of the kinase domain of EGFR and HER2 and has been used as an alternative treatment of HER2-positive breast cancer patients that developed resistance to Trastuzumab [8,9]. It has also been used in combination with chemotherapeutic compounds such as Capecitabine, and has been shown to reduce the risk of disease progression in women with advanced HER2-positive breast cancer who had received multiple previous treatments [17]. More recently, Lapatinib has been used in combination with letrozole (Femara) to treat postmenopausal women with Hormone receptor (HR) positive, HER2-positive metastatic breast cancer. This combination resulted in increased progression free survival in the HER2-positive population [14]. Gefitinib (Iressa) and Erlotinib (Tarceva), which are also tyrosine kinase inhibitors, have been used in treatment of patients with metastatic non-small-cell lung cancer. These drugs have been used in combination with chemotherapy and resulted in an improved and progression-free survivals [15,16]. Finally, Afatinib (Giotrif) is a novel ErbB family blocker that selectively blocks ErbB family members (EFGR, HER2, ErbB4 and ErbB3). Unlike Gefitinib and Erlotinib, Afatinib irreversibly (covalently) binds to proteins of ErbB family members and blocks their signaling pathways, thus promoting a sustained anti-proliferative activity [18,19]. This drug has been tested in several clinical trials and has been shown to extend progression free survival of patients with non-small cell lung carcinoma (NSCLC). However, this effect appears to be more beneficial to patients carrying EGFR del19 mutations [20]. Furthermore, and as Afatinib targets HER2, it is also being investigated for use in other HER2-positive cancers such as HER2-positive breast cancer [21].

#### 2.1.2. VEGFR-Targeted Therapy

This family of receptors, which binds VEGF, plays a key role in vasculogenesis and angiogenesis and is critical to tumor-induced new vascular formation [49]. Several studies have reported elevated levels of VEGFR in several cancers and these correlated with metastasis and poor prognosis [50,51,52]. A number of VEGFR inhibitors have been developed with the aim of reducing angiogenesis and lymphangiogenesis associated with cancer progression [49]. Sorafenib (Nexavar), a small molecule inhibitor of tyrosine protein kinase, has been used for the treatment of renal cell, liver and thyroid cancers. An improved progression-free survival following Sorafenib treatment was reported in patients with advanced renal cell cancer and nonresponsive thyroid cancer [22,23]. In patients with liver cancer, an improvement of median overall survival was reported [24]. Sunitinib (Sutent, SU11248) is another VEGFR protein tyrosine kinase inhibitor, which has been shown to improve overall survival of patients with renal cell cancer and gastrointestinal stromal tumor [25,26]. Besides the use of small molecule inhibitors to target VEGFR, a monoclonal antibody (Bevacizumab, Avastin) has been used in combination with chemotherapy to treat patients with metastatic colorectal carcinoma. This resulted in improvement of patients’ survival [27].

#### 2.1.3. PDGFR-Targeted Therapy

PDGF and PDGFRs have important functions in the regulation of cell growth and survival. Mutations within PDGFRα gene have been found in 5% of gastrointestinal stromal cancer (GIST). These mutations affect tyrosine kinase domains and juxtamembrane domain [53]. PDGFR genes were also involved in gene rearrangements found in certain leukemias [54]. In addition, amplifications of PDGFRα were reported in 5%–10% of glioblastoma multiforme, in oligodendrocytoma, esophageal squamous cell carcinoma and artery intimal sarcomas [55,56,57,58,59,60]. As for other dysfunctional RTKs, tyrosine kinase inhibitors have been developed to target directly PDGFR or as a secondary target. These small molecule inhibitors include imatinib, sunitinib, sorafenib, pazopanib and nilotinib. Imatinib (Gleevec), a well-known inhibitor of the oncogenic Bcr-abl fusion protein responsible for chronic myelogenous leukemia (CML), has been used to target PDGFR in gastrointestinal stromal tumors KIT positive. Although this treatment led to significant improvement of overall survival, many patients developed resistance to imatinib [28]. Other drugs such as sunitinib, soratinib, pazopanib and nilotinib were used to target multiple RTK receptors (e.g., PDGFR and VGFR) with the aim of inhibiting cell proliferation and angiogenesis to ensure maximum shrinkage of the tumor [29,30,31,32].

#### 2.1.4. FGFR-Targeted Therapy

Several mutations affecting FGFR genes have been reported in the literature [61]. Amplifications of FGFR1 and 2 have been found in breast cancer [62,63,64,65,66,67,68,69,70] and in gastric cancer where these mutations were associated with poor prognosis [71,72]. FGFR1 amplifications were found in bladder cancer, oral squamous carcinoma and ovarian cancer [73,74,75]. Point mutations that affect FGFR1, 2 and 3 lead to the increase of receptors or constitutive activations and were observed in cancer of the prostate, bladder, breast, brain, lung, uterus, stomach, head and neck, colon and malignant melanoma [76,77,78,79,80,81,82,83,84,85,86]. Chromosomal translocations involving FGFR genes generate oncogenic protein fusions that are present in several hematopoietic malignancies such as multiple myelomas and myeloproliferative disorder syndrome [87,88,89,90,91,92]. Although several small molecule inhibitors of the FGFR tyrosine kinase are currently in clinical development, these molecules also target other RTKs such as VEGFR, PDGFR and c-Kit [33,34,35,93,94,95,96,97,98,99]. Examples of these inhibitors include Brivanib (BMS-540215), a dual effect inhibitor of FGFR and VEGFR that has been shown to affect tumor growth in mouse models of human hepatocellular carcinoma (HCC) [33]. CHIR-258 (TKI-258), a multiple target inhibitor (VEGFR, PDGFR, FLT-3, c-Kit and FGFR), is an effective inhibitor of multiple myelomas harboring the translocation t (4, 14) (p16; q32) that expresses wild type or activated FGFR3 [34,35].

#### 2.1.5. MET-Targeted Therapy

MET is the receptor for the hepatocyte growth factor and is involved in cell growth, migration, invasion, metastasis and angiogenesis [100,101]. MET mutations and amplifications have been reported in many cancers such as neuroblastoma, glioblastomas, osteosarcomas, oesophageal and gastric colorectal cancers, multiple myelomas and T-cell leukemia. These alterations have been shown to be a driver of proliferation, invasion and metastasis and are associated with aggressive phenotype and poor prognosis [102,103,104,105,106,107,108,109,110,111,112]. Since the generation of the first c-MET inhibitor K252a [113], several inhibitors of MET have been tested clinically [114,115]. Among these inhibitors, SGX523 is a highly specific inhibitor of MET and has been shown to inhibit the growth of MDCK and A549 cells and GTL16 xenografts [36]. ARQ197 (ArQule) is also a MET-specific inhibitor that has been shown to inhibit the growth of the breast cancer cell line MDA-MB-231, the prostate cancer cell line PC3, the colon cancer cell line HT29 and the pancreatic cancer cell line PaCa2 [116,117]. Other inhibitors have a Broad spectrum kinase inhibitor effect such as MP470 which acts on MET, RET, KIT, PDGFR and FLT3 or XL880 and PF2341066 that act on VEGFR2 and ALK, respectively [114].

#### 2.1.6. c-KIT-Targeted Therapy

c-Kit also known as CD117 or Mast/Stem Cell Growth Factor Receptor is a cell surface receptor of SCF (Stem Cell Factor). C-Kit activation by SCF initiates the activation of downstream pathways that are involved in the regulation of multiple cellular processes such as proliferation, survival, cell migration, hematopoiesis, stem cell maintenance, melanogenesis and gametogenesis. Mutations in *c-KIT* result in SCF-independent activation of downstream signaling pathways associated with increased proliferation and cell survival, mostly found in leukemia, gastrointestinal stromal tumors (GIST), testicular germ cell tumor (TGCT) and melanoma. The majority of oncogenic c-Kit mutations are located in the juxtamembrane region (e.g., c-Kit^V560G^) or within the kinase domain (e.g., c-Kit^D816V^) [118]. The tyrosine kinase inhibitor Imatinib (Gleevec), a well-known inhibitor of the oncogenic Bcr-abl fusion protein, has been used to target the juxtamembrane domain of c-KIT in GIST patients. However, secondary mutations occur in other parts of the receptor (such as exon 17) that renders the tyrosine kinase resistant to the inhibition of imatinib [37,38,119]. Although newer c-Kit drugs have been developed (dasatinib and PKC412) to overcome the resistance to imatinib, these drugs have made little impact. This is mainly due to the lack of well-validated inhibitors of the forms of KIT that carry certain types of mutations [39].

### 2.2. The RAS/MAP Kinase Pathway

This pathway is a central player for a multitude of physiological and pathological cellular processes such as growth, proliferation, differentiation, migration and apoptosis [120]. Its activation by RTKs triggers a cascade of phosphorylation involving downstream kinases which leads to the phosphorylation of target proteins in the nucleus and cytoplasm. The first part of the cascade relies on the activation of at least one of the four major MAP kinases: ERK1 and 2 (ERK1/2), ERK5, p38, and JNK. ERK1 and 2 are probably the most studied MAP kinases. Receptor tyrosine kinases frequently engage Erk1/2 by recruiting the RAS guanine exchange factor Sos to the plasma membrane. This factor is constitutively associated with the protein adapter Grb2, which brings Sos in close proximity to the small GTPase RAS resulting in a nucleotide exchange from GDP to GTP, a change of Ras protein conformation and activation of the serine/threonine kinase Raf. The next step involves Raf phosphorylation of Mek1/2 which in turn phosphorylate and activate Erk1/2 [121] (Figure 1).

The ERK5 pathway is the least studied pathway among the MAP kinase pathways. However, its emerging role as an important player in the regulation of tumour migration and invasion refocused the “spotlight” on this pathway [122]. ERK5 (also known as BMK1) is activated by various stimuli such as oxidative stress, growth factors and oncogenes and plays an important role in cell proliferation, survival, differentiation and embryonic development of the vascular system [122,123] (Figure 1). The role of ERK5 in cell proliferation was demonstrated by *in vitro* expression of a dominant-negative form that resulted in preventing HeLa cells from entering the S phase of the cell cycle [124]. Similar results were observed in other cancer cell lines, re-enforcing its role as a regulator of cell proliferation [122]. ERK5 involvement in cell survival has been shown using *in vivo* and *in vitro* experimental approaches. The inactivation of the MEK5/ERK5 pathways using a mek5^−/−^ mouse model sensitized the mek5^−/−^ mouse embryonic fibroblast (MEFs) to osmotic-stress-induced apoptosis [125]. In another model (mouse tumour xenograft model), the induced deletion of ERK5, significantly reduced tumour volume and vascular density, that were mediated by the pro-proliferative and pro-survival factors RSK (p90 ribosomal S6 kinase) and rpS6 (ribosomal protein) [126]. *In vitro*, knockdown of ERK5 using siRNA, triggered apoptosis and reduced chemo-resistance of HL-60 acute myeloid leukemia cells [126]. ERK5 also appears to play a role in prostate cancer invasion and metastasis. High levels of expression of ERK5 correlated with the presence of bony metastases and less favourable disease-specific survival in prostate cancer patients. This expression was associated with increased expression of the extracellular matrix proteinase MMP9 [127]. The role of ERK5 in controlling cell differentiation has been shown by its negative control of macrophage differentiation through negative regulation of the expression of macrophage colony stimulating factor receptor (M-CSFR) [128]. Finally, the role of the ERK5 pathway in neoangiogenesis has been evidenced by target depletion of ERK5 in xenograft tumour models of B16F10 melanoma and LL/2 Lewis lung cancer and which resulted in reduced mass and vascular density of the tumours [126].

The Jun N-terminal kinase (JNK) and the p38 MAPK pathways’ family members, also called stress activated protein kinase pathways, function in cell context and cell type specific manner [129] (Figure 1). JNK1, JNK2 and JNK3 are encoded by *MAPK8*, *MAPK9* and *MAPK10*. Although JNK1 and JNK2 are ubiquitously expressed, JNK3 is mainly expressed in the brain and testis. These factors mediate their response through targeting AP1, a heterodimeric transcription factor composed of Jun and Fos family members and which plays an important role in several cellular processes including proliferation, differentiation and apoptosis. In human cancer, mutations affecting *MAPK9* resulted in JNK1 high expression in liver and prostate cancers, while mutations of MAPK10 that led to a loss of function was associated with brain tumours [130,131]. Interestingly, in mouse models, the function of JNK1 and JNK2 in regulating cell proliferation appears to be complex. For instance, mice knockout experiments of JNK1 and JNK2 resulted in confusing results. JNK1 appears to have a tumor suppressor function, whereas JNK2 functions as a tumour promoter [132]. Moreover, JNK1 knockout, unlike JNK2, significantly decreased HCC (hepatocellular carcinoma) in the DEN (diethylnitrosamine)-induced HCC mouse model [133]. In a similar manner, JNK2 but not JNK1 knockout, prevented skin cancer formation that was induced by DMBA (7,12-dimethylbenz[*a*]anthracene) and PMA treatments [134,135]. A possible explanation to this might be linked to their ability to interact with JUN, a regulator of cell cycle progression [136]. Finally, higher expression levels of MKK4 and MKK7, two JNK MAP kinase activators, have been associated with high-grade prostate cancer [132].

The p38 family comprises four isoforms (p38α, p38β, p38γ and p38δ) which could have overlapping functions [137]. Although p38α is expressed in most tissues, p38β, p38γ and p38δ appear to be expressed in specific tissues such as the brain, skeletal muscle and endocrine glands [138,139]. The p38 MAPK pathway regulates the phosphorylation of several transcription factors, such as p53, activating transcription factor 2 (ATF2), ElK1; and protein kinases, including MAPK activated kinase 2 (MK2; also known as MAPK2), mitogen- and stress-activated protein kinase1 (MsK1), MAP kinase-interacting serine/threonine kinase 1 (MNK1) and MNK2). This wide spectrum of activities allows the p38 pathway to negatively regulate cell cycle progression at the G1/S and G2/M phases of cell cycle progression. This tumor suppressive role has been studied using mice disrupted in *MEK3* and *MEK6* genes, where these mice exhibit increased tumorigenic potential and Ras-induced transformation [140]. Although the p38 MAPK pathway is involved in tumor suppression, increased expression of the phosphorylated form of p38 correlated with malignancy of different types of cancers [138]. This is consistent with previous reports that support a role of p38 MAPKs in epithelial-mesenchymal transition (EMT), a key event associated with cell migration, invasion and tumor cells’ extravasation [140,141]. In contrast, p38 MAPK pathway appears to play a role in the resistance to anoikis, another important event in tumor migration and spreading, as it allows cancer cells to survive following a loss of contact with the extracellular matrix or their neighboring cells [142]. Overall, the p38 MAPK pathway appears to be expressed in several types of cancers and in this regard constitutes a potential target for cancer therapies.

#### Targeting the RAS/MAP Kinase Pathway in Cancer

Most of cancer associated mutations affecting the MAP kinase signaling pathway involve mutations in *RAS* and *RAF* genes [143,144]. Mutations in RAS family of genes (*K-RAS*, *N-RAS*, *H-RAS*) have been found in several types of cancers [145]. High frequency of KRAS mutations are found in pancreatic, large intestine, biliary tract, small intestine, lung, endometrial and ovarian cancers. High frequencies of NRAS mutations are associated with melanoma, nervous system, hematopoietic and lymphatic, and thyroid cancers. HRAS mutations have a high frequency in salivary gland, urinary tract, cervix, upper aerodigestive tract, penis, prostate and skin cancers.

Mutations associated with RAF family (A-Raf, B-Raf, and C-Raf) are mainly associated with the BRAF gene and are found in a variety of cancers with high frequency in malignant melanoma, thyroid and colon carcinoma [146]*.* This mutation is single-base missense substitution of valine with glutamic acid at codon 600 (V600E) of the kinase domain and is prevalent in melanomas and papillary thyroid carcinomas [147,148]. Although low frequency, ARAF mutations are found in ovary and large intestine tumors and CRAF mutations in ovary and lung tumors [145].

**Figure 3:**
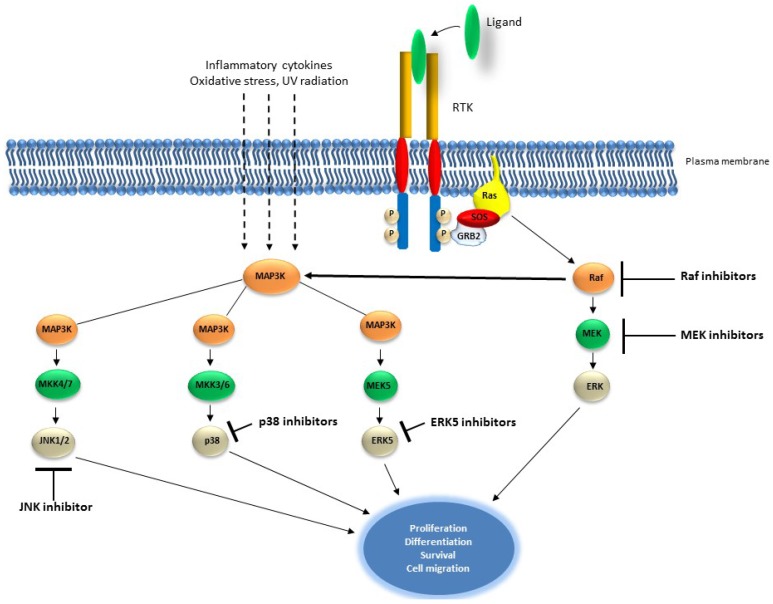
Schematic representation of components of the MAP kinase pathway targeted by small molecule inhibitors. The inhibitors (small molecule inhibitors and/or peptides) have been developed to target Raf, MEK, JNK1/2, p38 and ERK5.

Small inhibitor molecules are being developed to target primarily Mek and Raf in patients with different types of cancers [145] (Figure 3 and Table 2). Sorafenib, PLX4720, PLX4032 and GSK2118436 are drugs which are being used to target B-Raf^V600E^ in malignant melanoma and other advanced malignancies. Other chemical inhibitors such as LErafAON (NeoPharm) and ISIS 5132 are being used to target C-Raf in ovarian and breast cancers but also in other malignancies. MEK inhibitors such as CI-1040, PD-0325901, AZD6244, RDEA119/BAY 86-9766, GDC-0973/XL581 and AZD8330/ARRY-424704 which are also being tested clinically target MEK and for a wide variety of cancers, while others such as GSK1120212 target, in addition to MEK, C-Raf, B-Raf V600E and BRAF wild type [145]. Finally, inhibitors of the JNK proteins are being investigated for potential clinical use. These include the ATP-competitive JNK inhibitor SP600125 and JNK peptide inhibitor (D-JNKI-1), which showed promising results *in vitro* and *in vivo* tumor models [132] (Figure 3). P38 pathway inhibitors are mainly developed for treatment of diseases such as rheumatoid arthritis and Crohn’s disease, however, the inhibitor LY2228820 dimesylate produced significant inhibition of the tumors’ growth in *in vivo* models of melanoma, NSCLC, glioma, myeloma, and ovarian and breast cancers [149].

**Table 2:** Examples of MAP kinase and PI3K/AKT pathways’ factors targeted by molecular cancer therapies being used clinically or that are subject to clinical trials.

Target	Compound	Cancer	References
**MAP Kinase pathway**			
BRAF^V600E^	Sorafenib	Malignant melanoma	[143,144,145,146]
	PLX4720		
	PLX4032		
	GSK2118436		
C-RAF	LErafAON (NeoPharm)	Ovarian and Breast cancer	[143,144,145,146]
	ISIS 5132		
MEK	CI-1040	Various cancers	[143,144,145,146]
	PD-0325901		
	AZD6244		
	RDEA119/BAY 86-9766		
	GDC-0973/XL581		
	AZD8330/ARRY-424704		
**MAP Kinase pathway**			
C-RAF, MEK	GSK1120212	Various cancers	[143,144,145,146]
B-RAF^V600E^			
BRAF wild type			
**PI3K/AKT pathway**			
PI3K/mTOR	NVP-BEZ235	Various cancers	[150,151,152,153,154,155,156,157,158,159,160,161,162,163,164]
	BGT226		
	XL765/SAR245409		
	SF1126		
	GDC-0980		
	PI-103		
	PF-04691502		
	PKI-587		
	GSK2126458		

### 2.3. The PI3K/AKT Pathway

RTKs activation by growth factors, hormones, cytokines, neurotrophic factors and other extracellular signalling molecules trigger the activation of the lipid kinase PI3K, which phosphorylates phosphatidylinositol-4,5-bisphosphate (PIP_2_) on the plasma membrane and generates phosphatidylinositol-3,4,5-trisphosphate (PIP_3_) (Figure 1). The serine/threonine kinase Akt/PKB binds to PIP_3_, through its pleckstrin homology (PH) domain resulting in Akt translocation to the membrane and its partial phosphorylation by the phosphoinositide-dependent protein kinase 1 (PDK1) at Thr308. Akt is fully activated upon its phosphorylation at Ser473 by mTOR complex 2 (mTORC2) [165,166]. Following these series of activations, Akt phosphorylates several target proteins such as the glycogen synthase kinase 3α(GSK3α), mTOR, forkhead box O transcription factors (FoxO), MDM2, BCL2-interacting mediator of cell death (BIM) and BCL2-associated agonist of cell death (BAD), to facilitate cell survival and cell cycle entry [150,167]. Akt activation is negatively regulated by the Phosphatase and Tensin Homolog (PTEN), which dephosphorylates PIP_3_ preventing Akt translocation to the plasma membrane and thereby preventing its activation [150].

#### Targeting the PI3K/AKT Pathway in Cancer

Genetic mutations and amplifications affecting the different molecules associated with this pathway have been found in several cancers. The somatic mutation of *AKT1* isoform associated with the substitution of glutamic acid by a lysine at amino acid 17 (E17K) of Akt1 have been reported in human breast, colorectal, ovarian cancers and squamous cell lung carcinoma [168,169]. This mutation activates Akt1 by promoting its oncogenic localization to the plasma membrane, which stimulates downstream signaling resulting in cell transformation. Furthermore, this mutation is also found in *AKT3* gene and results in an oncogenic protein product (Akt3^E17K^) expressed in melanoma tumors [170]. Mutations in the catalytically active protein (PIK3CA/p100α) and the regulatory protein (p85α), which form the PI3K complex, have been reported in glioblastoma, ovarian, breast, colon, and endometrial cancers [171,172]. Mutations in the tumor suppressor PTEN, which negatively regulates Akt activation, have been reported in glioblastoma, melanoma, endometrial, prostate, breast and ovarian cancers [150]. These mutations are diverse and include insertion, substitutions and deletions. Genetic amplifications in *PIK3CA*, *AKT1* and the *AKT2* genes have also been reported. Other amplifications in the *PIK3CA* gene were found in squamous cell lung carcinoma, head and neck, cervical, gastric, and oesophageal cancers [173,174,175]. Amplifications in the *AKT1* gene were associated with gastric cancer and in *AKT2* gene with head and neck, pancreatic, ovary and breast cancers [173,176,177,178,179].

**Figure 4:**
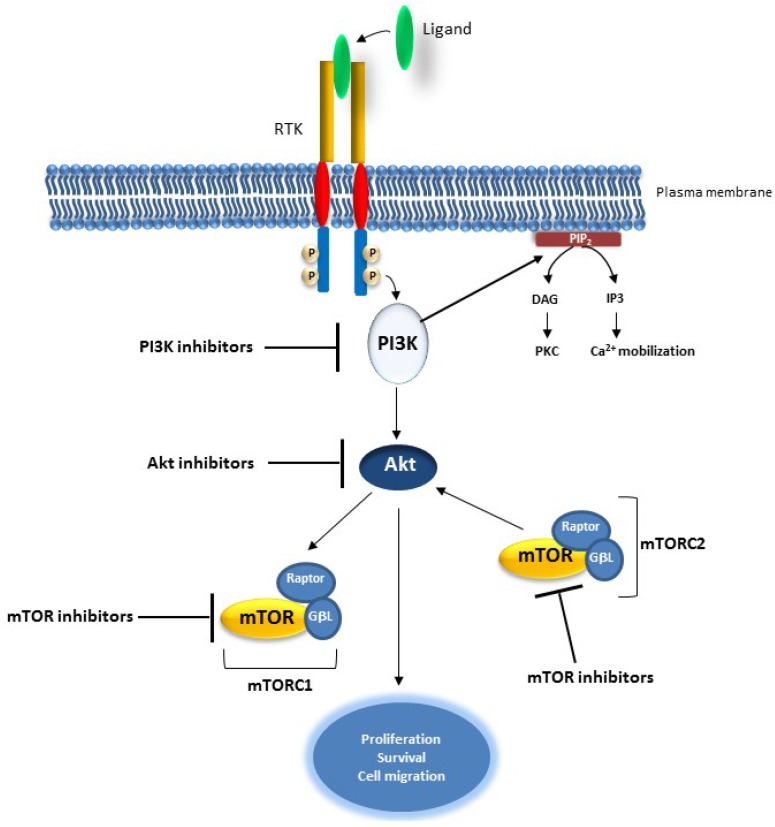
Schematic representation of components of the PI3K/AKT pathway (PI3K, Akt and mTOR) targeted by small molecule inhibitors.

Several small molecule inhibitors of the PI3K/AKT pathway have been developed (Figure 4 and Table 2). Some of these drugs have a dual inhibitor activity or target a specific component of this pathway. NVP-BEZ235, BGT226, XL765/SAR245409, SF1126, GDC-0980, PI-103, PF-04691502, PKI-587, and GSK2126458 have a dual activity toward PI3K and mTOR [153]. These inhibitors, some of which are being tested clinically, target the isoforms of PI3K and the ATP-binding sites of mTORC1 and mTORC2 [152,153]. Small inhibitor molecules have been developed to target specifically PI3K; among them XL147, PX866, GDC0941, BKM120, CAL101 (targets p110δ) are at early or late clinical development for patients with advanced solid tumors and lymphomas [154,155,156,157,158,159,160]. Small molecule inhibitors targeting Akt which include Perifosine, GSK690693, VQD002 and MK2206 are also being tested clinically [150,160,161,162]. Finally, OSI027 and AZD8055, which target and inhibit the catalytic site of mTOR, have shown clinical utility in certain cancers [163,164].

## 3. Concluding Remarks

Although significant progress has been made in developing small molecule inhibitors and monoclonal antibodies that target components of the RTK signaling pathways in cancer, substantial challenges prevent rapid and efficient therapies. In this regard, an important obstacle remains in the capacity of cancer cells to adapt to these inhibitors by developing resistance through the emergence of additional mutations. Therefore, complementary inhibitors have to be developed to overcome this resistance. A combination of inhibitors which target RTK, components of the MAP kinase (MEK or Raf inhibitors) or PI3K/AKT may have a better effect in cancer patients’ treatment. Finally, cancers have heterogeneous populations of cells which may react differently to those inhibitors and might play a role in chemo-resistance and cancer initiation and progression following chemotherapeutic treatments. Cancer stem cells are a well-known example of chemo-resistance that leads to relapse in cancer patients. For instance, malignant melanoma stem cells express the ATP-binding cassette transporter ABCB5 which plays an important role in drugs efflux, and, thereby, may attenuate the therapeutic efficiency of the inhibitors used in cancer therapy [180]. Therefore, a better understanding of tumor cellular heterogeneity would result in better therapeutic design and more efficient drugs.
